# Long-Term Effects of Abuse in Later Life Perpetrated by Family Members

**DOI:** 10.1093/geroni/igaa057.1438

**Published:** 2020-12-16

**Authors:** Naomi Meinertz, Pi-Ju Liu, Ron Acierno

**Affiliations:** 1 Iowa State University, Ames, Iowa, United States; 2 Purdue University, West Lafayette, Indiana, United States; 3 UT Health Sciences Houston, Houston, Texas, United States

## Abstract

Abuse in later life could potentially lead to lower levels of social support, especially when perpetrated by family members who are charged with protecting the older adult in their care. Using both waves of the National Elder Mistreatment longitudinal data (wave one collected in 2008 and wave two in 2015; N=774), long-term effects of abuse (i.e., physical, emotional, sexual, and financial) on levels of social support, physical health, and clinical depressive symptoms for respondents at or above the age of 60 years were analyzed. A multivariate analysis of variance showed that respondents abused at wave one (n=261) by a family member (B=-0.55, p≤0.001), a spouse or ex-partner (B=-0.349, p=0.02), or a non-relative or stranger (B=-0.301, p=0.026) had lower levels of social support eight years later at wave two. Those abused by a family member at wave one also experienced higher levels of depressive symptoms at wave two (B=-0.187, p=0.01). Perpetrator type did not predict general health at wave two. These results emphasize the long-term impact of abuse on the lives of older adults and highlight the importance trusted relationships, such as with family members, have on older adult health and wellbeing.

